# Supplementary material for: Causal Relationship between Aging and Anorexia Nervosa: A White-Matter-Microstructure-Mediated Mendelian Randomization Analysis
Source: Biomedicines. 2024 Aug 16;12(8):1874. doi: 10.3390/biomedicines12081874 (PMC11351342; doi:10.3390/biomedicines12081874)
Supplement: Supplementary file 1 [file biomedicines-12-01874-s001.zip › Supplementary Figure/Figure S4/Singe SNP plot of MR analysis for TL to ubm-b-2064.pdf]

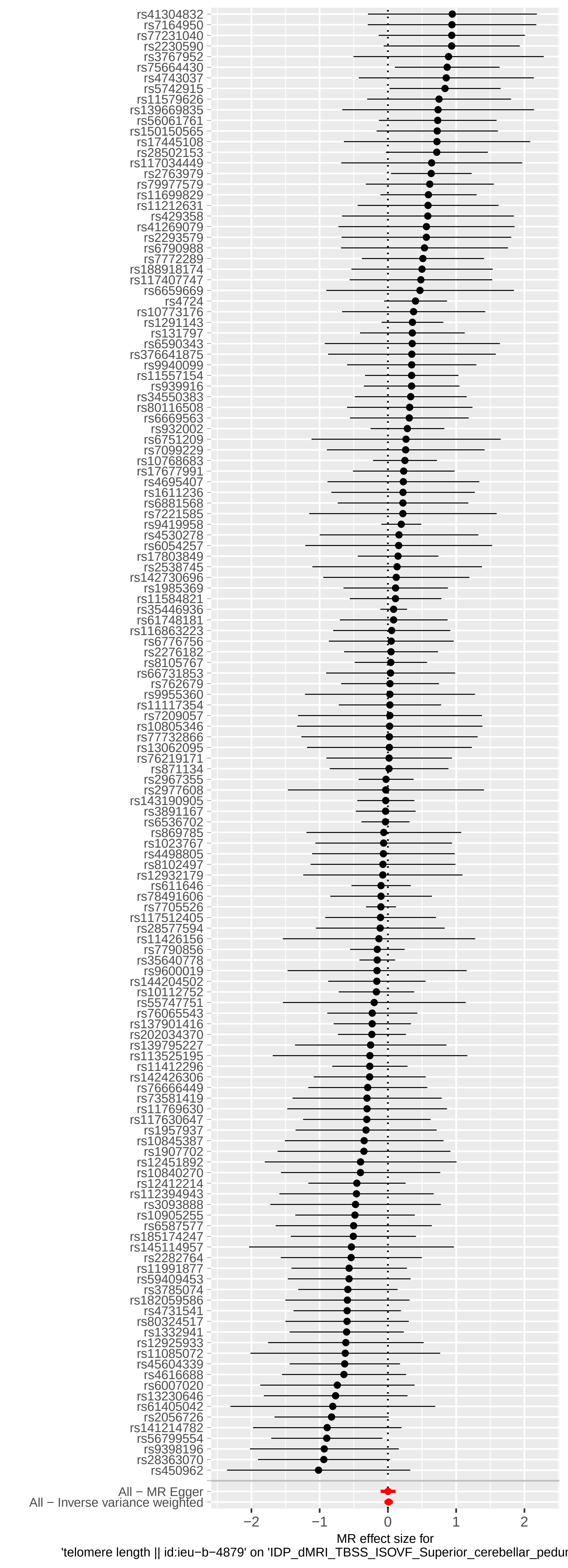
rs41304832
rs7164950
rs77231040
rs2230590
rs3767952
rs75664430
rs4743037
rs5742915
rs11579626
rs139669835
rs56061761
rs150150565
rs17445108
rs28502153
rs117034449
rs2763979
rs79977579
rs11699829
rs11212631
rs429358
rs41269079
rs2293579
rs6790988
rs7772289
rs188918174
rs117407747
rs6659669
rs4724
rs10773176
rs1291143
rs131797
rs6590343
rs376641875
rs9940099
rs11557154
rs939916
rs34550383
rs80116508
rs6669563
rs932002
rs6751209
rs7099229
rs10768683
rs17677991
rs4695407
rs1611236
rs6881568
rs7221585
rs9419958
rs4530278
rs6054257
rs17803849
rs2538745
rs142730696
rs1985369
rs11584821
rs35446936
rs61748181
rs116863223
rs6776756
rs2276182
rs8105767
rs66731853
rs762679
rs9955360
rs11117354
rs7209057
rs10805346
rs77732866
rs13062095
rs76219171
rs871134
rs2967355
rs2977608
rs143190905
rs3891167
rs6536702
rs869785
rs1023767
rs4498805
rs8102497
rs12932179
rs611646
rs78491606
rs7705526
rs117512405
rs28577594
rs11426156
rs7790856
rs35640778
rs9600019
rs144204502
rs10112752
rs55747751
rs76065543
rs137901416
rs202034370
rs139795227
rs113525195
rs11412296
rs142426306
rs76666449
rs73581419
rs11769630
rs117630647
rs1957937
rs10845387
rs1907702
rs12451892
rs10840270
rs12412214
rs112394943
rs3093888
rs10905255
rs6587577
rs185174247
rs145114957
rs2282764
rs11991877
rs59409453
rs3785074
rs182059586
rs4731541
rs80324517
rs1332941
rs12925933
rs11085072
rs45604339
rs4616688
rs6007020
rs13230646
rs61405042
rs2056726
rs141214782
rs56799554
rs9398196
rs28363070
rs450962
All − MR Egger
All − Inverse variance weighted
−2
−1
0
1
2
MR effect size for
'telomere length || id:ieu-b-4879' on 'IDP_dMRI_TBSS_ISOVF_Superior_cerebellar_pedur
